# Barriers and facilitators to facility HIV self-testing in outpatient settings in Malawi: a qualitative study

**DOI:** 10.1186/s12889-021-12213-6

**Published:** 2021-12-02

**Authors:** Misheck Mphande, Paula Campbell, Risa M. Hoffman, Khumbo Phiri, Mike Nyirenda, Sundeep K. Gupta, Vincent Wong, Kathryn Dovel

**Affiliations:** 1 Partners in Hope, Box, 302 Lilongwe, Malawi; 2 Division of Infectious Diseases, David Geffen School of Medicine, University of California Los Angeles, Los Angeles, CA USA; 3 Office of HIV/AIDS, United States Agency for International Development, Washington D.C, USA

## Abstract

**Background:**

Facility HIV self-testing (HIVST) within outpatient departments can increase HIV testing coverage by facilitating HIVST use in outpatient waiting spaces while clients wait for routine care. Facility HIVST allows for the majority of outpatients to test with minimal health care worker time requirements. However, barriers and facilitators to outpatients’ use of facility HIVST are still unknown.

**Methods:**

As part of a cluster randomized trial on facility HIVST in Malawi, we conducted in-depth interviews with 57 adult outpatients (> 15 years) who were exposed to the HIVST intervention and collected observational journals that documented study staff observations from facility waiting spaces where HIVST was implemented. Translated and transcribed data were analyzed using constant comparison analysis in Atlas.ti.

**Results:**

Facility HIVST was convenient, fast, and provided autonomy to outpatients. The strategy also had novel facilitators for testing, such as increased motivation to test due to seeing others test, immediate support for HIVST use, and easy access to additional HIV services in the health facility. Barriers to facility HIVST included fear of judgment from others and unwanted status disclosure due to lack of privacy. Desired changes to the intervention included private, separate spaces for kit use and interpretation and increased opportunity for disclosure and post-test counseling.

**Conclusions:**

Facility HIVST was largely acceptable to outpatients in Malawi with novel facilitators that are unique to facility HIVST in OPD waiting spaces.

**Trial registration:**

The parent trial is registered with ClinicalTrials.gov, NCT03271307, and Pan African Clinical Trials, PACTR201711002697316.

## Introduction

In 2019, there were 20.7 million people living with HIV (PLHIV) in sub-Saharan Africa (SSA) [1]. However, more than 25% of adults in the region still do not know their serostatus [2]. In Malawi, only 42% of the adult population tested for HIV within the past 12 months [3], with 35% of men having never tested for HIV [4, 5]. Additionally, there are an estimated 33,000 new HIV infections in Malawi per year requiring diagnosis and treatment [6]. Facility-based provider-initiated testing and counseling (PITC) remains the primary strategy for testing [7], although it continues to have limited reach in busy outpatient settings [8, 9].

HIV self-testing (HIVST) can improve PITC among outpatients in high-burden settings (i.e., facility HIVST) and studies have shown that HIVST is highly acceptable [10–12]. We previously conducted a randomized control trial (RCT) and found that facility HIVST can increase testing among adult outpatients by 200% compared to standard PITC [2] and is cost-effective in routine settings by capitalizing on the existing health infrastructure [13]. Although there is insufficient data on barriers and facilitators for facility HIVST implementation, there is extensive literature on community HIVST [11]. Known facilitators for community HIVST include increased privacy, perceived autonomy and confidentiality of test results, perceived convenience, and minimal time required to test [14–16]. These benefits are believed to largely remove demand-side barriers to traditional testing strategies, including travel time and cost, fear of unwanted disclosure, and long clinic wait times [15]. Reported barriers are few and include doubts or misconceptions about the accuracy of HIVST kits and individuals’ ability to use and interpret HIVST kits unsupervised [11, 17].

The above-mentioned barriers and facilitators may differ for facility HIVST since this strategy reduces the privacy as compared to community HIVST (testing at the facility is often done in public areas such as waiting spaces), but may also provide immediate linkage and prevention services. Given this gap in evidence, we analyzed qualitative data collected as part of a parent facility HIVST trial to examine adult perceptions of HIVST in outpatient departments, and understand what components of facility HIVST strategies are crucial for intervention scale-up.

## Method

### Design and setting

Our parent cluster-randomized trial aimed to assess the impact of facility HIVST among adults attending outpatient departments (OPD) in Malawi. Outpatient departments are the primary entry point to most service delivery strategies in Malawi and offer a range of services from acute care for injuries and general illness to treatment for sexually transmitted infections. The parent trial compared the impact of facility HIVST against standard PITC and optimized PITC, whereby additional training and job aids were given to health workers, and morning HIV testing was offered to all outpatients [13]. In the study (a full description is published elsewhere) [13, 18], 15 facilities were cluster randomized 1:1:1 to three arms: (1) standard PITC, (2) optimized PITC, and (3) facility HIVST whereby OraQuick ADVANCE HIV I/II self-testing kits [19] were distributed during adult outpatient services in waiting spaces. In this paper, we include data from the facility HIVST arm that was implemented in 5 randomly selected facilities including a district hospital (*n* = 1); rural hospital (n = 1), non-profit hospital (*n* = 1); and health center (*n* = 2). Eligibility criteria for participation in the parent study included: > 15 years of age and having received outpatient services on the day of recruitment.

### Intervention

**Fig. 1:**
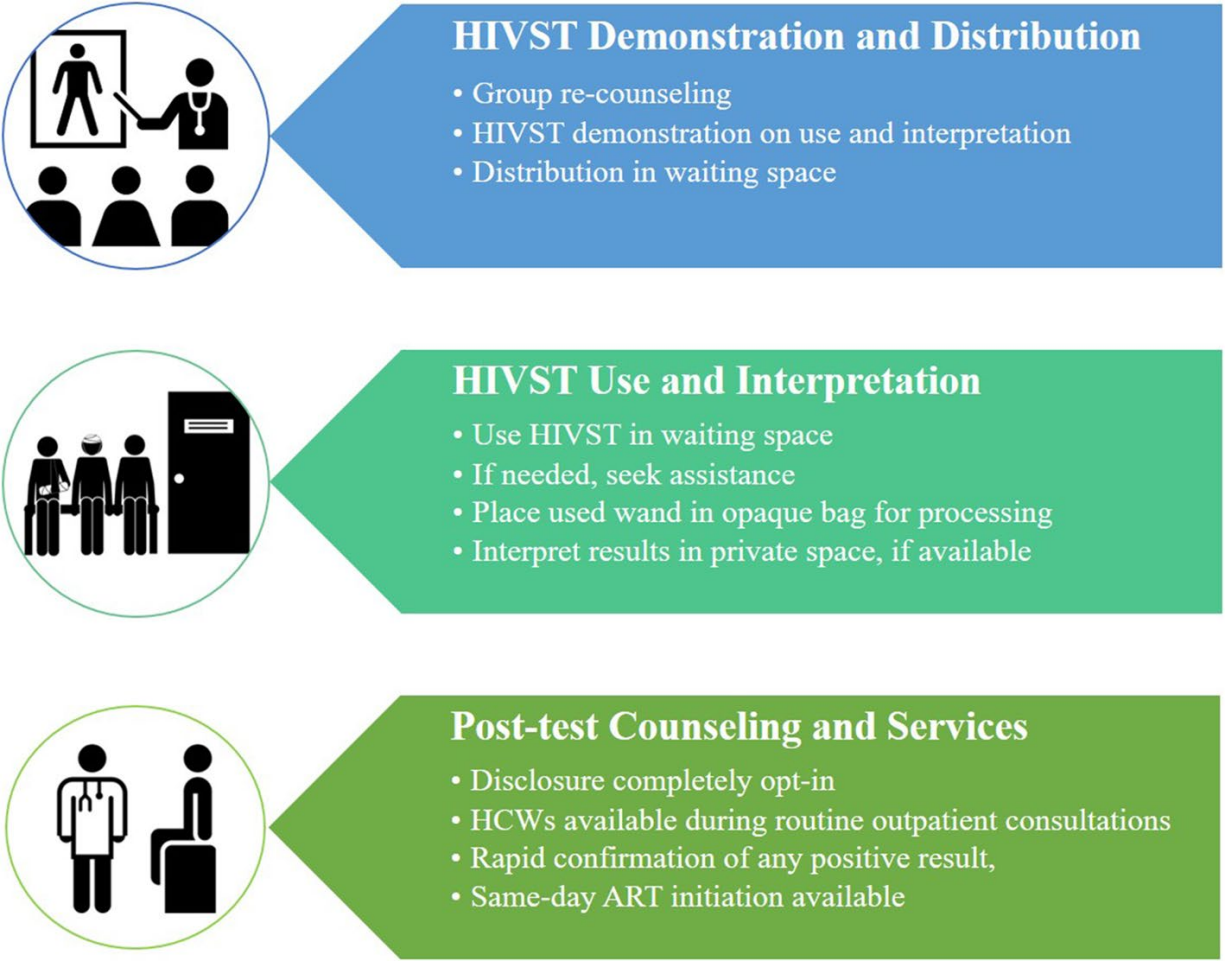
Overarching components of the intervention

Facility HIVST included three overarching components: (1) HIVST demonstration and distribution; (2) HIVST use and interpretation; and (3) post-test services and counseling (Fig. 1). A 10-min health talk about the importance of HIV testing and a 15-min demonstration on HIVST kits, followed by kit distribution, was conducted in outpatient waiting spaces. HIVST was offered one-on-one in order to create a true opt-out service; however, distribution was in open waiting spaces – private rooms were not used for demonstration and distribution activities.

Outpatients took and used the kit in the OPD waiting space prior to receiving routine outpatient services. HIVST users were strongly encouraged to *not* look at their HIVST result in the public waiting space. For privacy in the waiting space, HIVST kits were stored in an opaque bag for processing. Private spaces (rooms or kiosks near the OPD) were provided for HIVST interpretation prior to receiving routine outpatient services. Trained study staff were available to provide assistance with HIVST use as needed. Finally, post-test counselling and referral to additional HIV services were available upon request – outpatients were not asked HIVST results by study staff. Outpatient HCWs were encouraged to ask about HIVST test results, but disclosure was not mandatory.

### Data collection

For the parent trial, exit surveys were conducted with a 5885 sample of outpatients. In the HIVST arm, 2097 surveys were completed and provided the sampling frame for our current sub-study. Two forms of qualitative data are included in the current sub-study: in-depth interviews with outpatients (*n* = 57) and observation journals collected by research assistants who observed OPD waiting spaces in participating facilities (81 typed pages from 38 observation days).

#### In-depth interviews

Participants in the parent trial were randomly selected for an in-depth interview using an electronic randomization tool. Eligibility criteria for the additional in-depth interviews included: 1) > 15 years of age; 2) received outpatient services on the day of recruitment; 3) enrolled in the larger trial; and 4) exposed to the facility HIVST intervention (defined as heard of HIVST while at the health facility that same day). All respondents provided oral informed consent prior to data collection. All interviews were conducted the same day in a private location within the health facility and all participants were given the equivalent of $4 for their time.

A guide was developed based on existing literature [17, 18] and included questions about previous experiences with HIV testing services, experience with facility HIVST that day, barriers and facilitators to facility HIVST, and recommendations (see Appendix A for the Interview Guide). Respondents and interviewers were matched by gender (i.e., man interviewing a man). Interviews were conducted in the local language (Chichewa) and lasted approximately 40 min.

#### Observational journals

Observational journals were collected by research assistants during the parent intervention. Observational journals are a form of local ethnography [20, 21] whereby research assistants observed facility waiting spaces where HIVST kits were distributed and used. At the end of each day, research assistants wrote what they observed, recalling as much detail as possible about the content and context of client and client-provider interactions. Observational journals are well suited to capture practices within a health institution, documenting what people say and how they interact with others, which can be different from what they report in traditional research settings [21]. While journals cannot capture all events within one setting, they provide an important overview of common interactions and conversations within routine settings [22].

Research assistants made observations and described the content and context of interactions within OPD waiting spaces over 38 days (resulting in over 81 typed pages of observational journals). Researchers were instructed to document interactions on the following topics: (1) distribution of HIVST; (2) conversations between clients regarding HIVST; (3) potential or observed unwanted disclosure, including others asking about one’s test result; and (4) discussed or observed concerns regarding facility HIVST. Two of the five facilities were excluded from analysis due to research staff shortages and incomplete data.

### Data analysis

In-depth interviews and observational journals were transcribed and translated into English (if needed). All transcripts were coded using inductive and deductive strategies [23]. The same coding techniques and codebook were applied to both in-depth interview and observational journal data. Deductive codes were developed based on existing literature [23], and were the basis for the original codebook. MM and PC piloted the codebook on 5 in-depth interviews and 4 observational journals. Inductive codes were added as needed. Co-authors reviewed codes and resolved any disagreements to make a final codebook. Two co-authors (MM and PC) coded all transcripts using Atlas.ti. Coded data were analyzed using constant comparison methods [24]. We compared dominant and emerging themes by HIVST users and non-users and by sex, reporting any differences.

### Ethical approval

All study activities were approved by the Institutional Review Board at University of California Los Angeles (Protocol number 17–000109) and the National Health Sciences Review Committee in Malawi (Protocol number 1664). All data collection efforts were carried out in accordance with relevant guidelines and regulations.

## Results

We conducted in-depth interviews with 57 adult outpatients who were offered HIVST at the OPD between Sept 12, 2017 and Feb 23, 2018. Twenty-six in-depth interview respondents were male and 31 were female. Among respondents interviewed, 46 reported using HIVST during their OPD visit (users), while 11 respondents heard about HIVST but did not use a kit (non-users). (See Table 1).

**Table 1:** Demographics of outpatients who participated in an in-depth interview, including HIVST users and non-users (*n*=57)

Variable	Users	Non-Users	Total
Sex			
*Male*	*20*	*6*	26
*Female*	*26*	*5*	31
Status			
*HIV-positive*	*6*	*N/A*	6
*HIV-negative*	*40*	*N/A*	40
Region			
*South*	*28*	*8*	36
*Central*	*17*	*4*	21
Facility Type			
*Non-Profit Facilty*	*5*	*2*	7
*District Hospital*	*7*	*6*	13
*Rural Hospital*	*17*	*5*	22
*Health Center*	*11*	*4*	15

Among non-users, the primary reason reported for not using HIVST was having tested recently, often within the past three months (see Table 2). Notably, only one client reported that he did not test because he did not trust the accuracy of HIVST.

**Table 2:** Reasons for not using facility HIVST among non-users (*n*=11)^a^

Reasons for not testing	Total
Tested within 3 months	5
Perceived low risk of HIV	2
Too sick to test (poor health)	1
Not prepared to test	1
Doubt about kit accuracy	1
Time required to test	1

^a^based on responses to open-ended questions in the interview

**Fig. 2:**
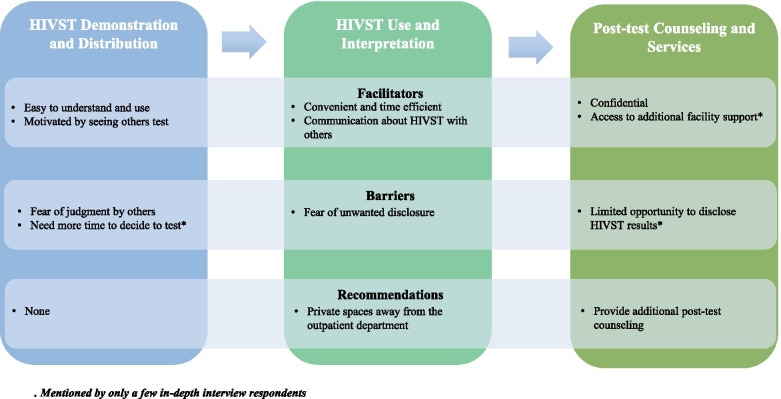
Facilitators, barries, and recommended changes to each components of the facility HIVST intervention

Below, we present results according to each component of the facility HIVST intervention: (1) health talk/demonstration and distribution; (2) HIVST use and interpretation; and (3) provider disclosure and linkage to additional care (see Fig. 2). We present respondent perceptions of each component, including the most salient themes around the benefits and challenges to facility HIVST, distinguishing between users and non-users, and men and women.

### HIVST demonstration and distribution

#### Easy to understand and use

None of the interview respondents had ever used HIVST prior to the current intervention, however most reported that they had enough education about how to use HIVST and that the demonstrations were clear. All respondents could explain how to use and interpret HIVST.

*I was thinking that maybe the procedure is a bit difficult because when you do things for the first time, it seems as if it's difficult. But when we followed [directions], it is the shorter method and easy to understand and very cheap [fast]. (Male, HIVST User, Health Center)*

#### Motivated by seeing others test

Over half of HIVST users explained that open, group distribution inspired them to test. The open layout meant that anyone in the waiting space could easily see who accepted a HIVST kit. Respondents described being “carried away” or persuaded by seeing others take HIVST kits or discussing HIVST with others in the waiting space. Other outpatients who rapidly took up testing acted as positive reinforcement, encouraging respondents who were initially unsure to test. More females reported having open conversations about HIVST; however, more men viewed their conversations as crucial to their decision to test.

*What the HCWs were explaining all this year, I wasn’t taking to heart, … . [but] With the way they explained [HIVST] I got carried away. For the first time I tested. I saw that people were getting tested so I thought I should also do the test … they [other clients] just said, ‘Let us get them [HIVST].’ We were just talking to each other which means they also got carried away like I did. (Male, HIVST User, Non-Profit Facility)*

*Since I was also looking at my friends [outpatients] self-testing then I thought, ‘I too should test instantly’ [right away]. (Female, HIVST User, Health Center)*

Observational journals frequently documented similar stories of group distribution inspiring testing.

*They [outpatients] saw other people carrying the self-test kits … A certain man [outpatient] answered, ‘It is a new testing method.. If you are a patient and you are ready to test yourself, tell the owners [study staff] to give you the test kits. They will teach you how to use them’ … All three women got the kits and used them.* (*Observational Journal, Rural Hospital*)

#### Fear of Judgement by others

Less than a quarter of interview respondents believed group distribution lacked privacy, hindering HIVST uptake. More men than women reported lack of privacy as a barrier to uptake. Some respondents also reported feeling “shy” or uncomfortable receiving a kit because others would question their HIV status or assume they were sexually promiscuous.

*When I was receiving it [HIVST], many people were looking at me, and I could feel shyness on my face. It was difficult to stay happy. But all-in-all, I tried getting the kit and used it. (Male, HIVST User, District Hospital)*

Observational journals documented two instances where clients refused HIVST because they felt uncomfortable taking kits in waiting spaces. Study staff heard one male outpatient explain:

*I cannot ask for the kit here and use it in the presence of all these people. Had it been that they are calling us one-by-one in a private room, I could have managed to ask for the test kit. (Rural Hospital Observational Journal 3, 14:9* ***)***

### HIVST use and interpretation

#### Convenient and fast

The majority of interview respondents reported that using HIVST while waiting for routine outpatient services was fast and efficient when compared to routine blood-based HIV testing with PITC. Many outpatients positively described facility HIVST as allowing them to “kill two birds with one stone.”

*I felt it would be better I do both [OPD services and HIVST] at once to kill two birds with one stone ... by the time I am going to meet the doctor I am also at the same time knowing my HIV status with this [HIVST] kit. (Male, HIVST User, Rural Hospital)*

When asked if he would have tested for HIV that day if HIVST was not available, one man replied:

*I shouldn’t lie, I wouldn’t have gotten tested … I came because of malaria. But when I also heard that there is something else [HIVST] and the way is not hard [to use] I just said, ‘Aah I should rush [to test].’ (Male, HIVST User, District Hospital)*

#### Peer support on how to use HIVST

Over half of respondents reported discussing how to use HIVST with others in the waiting space. However, about a quarter of respondents stated that they or other outpatients had questions or needed additional help regarding HIVST. Some respondents understood the HIVST process better than others and would clarify kit use for others. Other respondents simply discussed the benefits of HIV testing and encouraged each other to continue testing in the future.

*The topic we [outpatients] were discussing was all about [HIVST] that it is good … in the process of chatting the 20 minutes time [for processing] was done and we were very open people. (Male, HIVST User, Rural Hospital)*

#### Fear of unwanted disclosure

Despite positive experiences expressed by most respondents, nearly a quarter of interview respondents desired more privacy for interpreting HIVST results. A handful of respondents feared other people in the waiting space may try to look at their HIVST kit results (referred to as “peeping”), while a few others described other outpatients either trying to look at their test result or directly asking what their HIVST result was.

*Because it was an open place, I had worries that when opening to see the results my friends will be peeping to see. (Female, HIVST User, Rural Hospital)*

Journals also recorded a handful of instances where outpatients were afraid that others would make educated guesses or assumptions about their HIV status based on body language or facial expressions after leaving spaces for interpretation of results. Limited available private space in clinics meant that most facilities relied on standing booths for private spaces to interpret HIVST results. While the booths themselves were private, located either in OPD waiting spaces against a wall so no one could see the interpretation space or outside the waiting space, others in the waiting space could see individuals walking to or from the interpretation booths, prompting onlookers to make assumptions about one’s HIV status. One journal reported:

*‘We [OPD clients] are chatting and smiling each other here because I know nothing about my status. But once I read my [HIVST result], my face will change [when I leave the booth]. I can even cry here... Those who saw me receiving the kit will automatically know that I am crying because I have read my test results, and it will mean to them [they will assume] that I am HIV positive.’ (Observational Journal, Non-Profit Facility)*

Due to fear of unwanted disclosure, a quarter of outpatients interviewed recommended having private rooms away from the OPD waiting space for HIVST interpretation, as rooms were believed to be more private than portable interpretation booths used by the intervention.

*Maybe you should have a separate room. For those who are open, [they] can get the kit there [waiting space], and for those who are shy to do it in public, [they] can go to that room. (Female, HIVST Non-User, Rural Hospital)*

### Post-test counseling and services

#### Autonomy

HIVST users liked that they had control over whether or not to disclose their HIVST result to HCWs. The desire to not disclose largely stemmed from a reported lack of trust in HCWs’ ability to maintain confidentiality, while some respondents simply appreciated not having the anxiety related to waiting for someone else to interpret their test results.

*I didn’t trust those people [HCW’s]. Since they were telling you [your] results, it is possible they could tell other people that, “Did you see the one who just went out there? Results came out HIV positive!” It’s something so disappointing and pathetic. You can have bad ideas that you should not do it [test] again. So I think this way [HIVST] is very good because you are the only one who can know your results … It’s all up to you. (Female, HIVST User, Non-Profit Facility)*

#### Limited opportunity to disclose HIVST results

While most interview respondents did not want to disclose their HIVST results to HCWs, they also said they had no opportunity to do so during routine outpatient consultations. The vast majority of respondents reported that HCWs did not ask about HIVST results, and clients did not bring it up. Respondents stated that they felt uncomfortable initiating conversations about HIVST because clinicians seemed too busy.

*I didn’t discuss with anyone … He [HCW] asked what’s wrong, I told him I am having a stomach problem … I had it [HIVST kit] in my hands, but he didn’t ask anything. (Female, HIVST User, Health Center)*

However, only two respondents indicated that the lack of engagement by HCWs left them with unanswered questions or concerns.

#### Additional post-test counseling

In light of minimal disclosure to HCWs, about a quarter of HIVST users recommended additional post-test counseling in facility HIVST. Most, however, wanted counseling without having to disclose their own test result. Client suggestions included having follow-up group post-counseling sessions with everyone in the outpatient waiting spaces (similar to the pre-counseling and HIVST demonstration), or one-on-one post-counseling during the outpatient consultation whereby the provider would provide broad post-test messaging for those who did not want to disclose. Most respondents suggested additional information on prevention strategies and the benefits of immediate ART initiation – including treatment as prevention –to motivate others to test.

*They should explain deep [post-counseling]. People were left in suspense. They [health care workers] should go into details, … if you see your results whether positive or negative you must meet the doctor. (Male, HIVST User, Non-Profit Facility)*

## Discussion

Facility HIVST was highly acceptable to both female and male adult outpatients in Malawi. Facility HIVST retained some of the most important benefits of traditional HIVST identified throughout the literature – HIVST was still seen as easy to use, convenient, required minimal time, and provided confidential results [16], although there were some concerns about privacy of distribution and use. We found that the group-style distribution of HIVST also resulted in additional facilitators to HIV testing usually not associated with HIVST, such as increased motivation to test due to seeing others test, being able to directly communicate with others about HIVST, and receiving immediate support from facility staff.

Many respondents in our study described being motivated by seeing other people test. The visible distribution of kits in waiting spaces helped to create a critical mass of outpatients testing, normalizing testing behavior in this particular setting. Similar arguments have been made for HIVST in other settings [25] and other stigmatized services that benefit from opt-out strategies [26]. However, frequent and highly motivating health talks and HIVST demonstrations in OPD are likely required in order to create excitement for HIVST and achieve a high coverage.

While group distribution acted as a facilitator for many respondents, it also served as a barrier for others. For some, group distribution discouraged testing because they believed other outpatients would see them test and judge them, making assumptions about their sexual risk behavior and HIV status. Similar findings have been reported in other studies, noting that private testing areas are essential in order to avoid stigma associated with taking up HIV testing [27, 28]. A handful of respondents were also concerned that using HIVST in waiting spaces would lead to potential coercion or unwanted status disclosure to other outpatients. Private spaces for HIVST distribution and use would mitigate these concerns, however, infrastructure constraints remain a major concern for local health facilities and may not be addressed without extensive investment.

We found that nearly half of respondents reported talking with other outpatients about how to use HIVST and about HIV more broadly. Even with a HIVST demonstration, some outpatients were still unclear on exactly how to use the kit and found other outpatients helpful in guiding them through the process. Other HIVST interventions vary in levels of support provided, with some offering assisted HIVST use [29] and others, such as secondary distribution of HIVST, offering no additional support [30]. HIVST interventions should maximize opportunities to support correct HIVST use to ensure optimal testing. Our study shows opportunities for support do not have to be exclusively from HCWs – other community members who have seen HIVST demonstrations may be able and willing to provide support.

Nearly all respondents believed they had little opportunity to disclose HIVST result to HCWs in OPD settings, largely because HCWs did not directly ask respondents about their results (disclosure was completely opt-in) and outpatients were not comfortable initiating the conversation. As a result, HCWs rarely offered post-test counseling, which was highly desired by respondents in the study, regardless of their HIVST result. Other studies find that disclosure is highest when individuals believe they will receive support from HCWs, and lowest when they expect blame and discrimination from HCWs [31]. Future HIVST interventions should foster a welcoming environment where HCWs actively ask about HIVST results and provide a safe space for post-test counseling.

Even though very few respondents disclosed their HIVST result to a HCW at OPD, many perceived the ability to immediately self-initiate connection (link) to additional HIV services at that same health facility as a major benefit to facility HIVST. Our parent trial on facility HIVST found that 79% of those who tested HIV-positive with HIVST initiated ART within 3-months, but only 7% disclosed their HIV-positive status to a HCW in OPD department. The vast majority went directly to the ART department within that same facility to link themselves to HIV care [13]. The fact that many HIVST users self-referred to the ART clinic for immediate treatment initiation may reflect a continued autonomy and ownership over one’s own health care that is promoted through self-testing. Further, situating HIVST strategies within OPD, which is on the same campus as ART services, may facilitate linkage as logistical barriers to initiation are already overcome, since individuals are already at the facility for acute or routine care. Other HIVST interventions within community settings report lower rates of linkage and ART initiation [32–35]. Future HIVST interventions should consider additional strategies to further reduce gaps to linkage to HIV treatment services.

This study has several limitations that should be noted. Facility HIVST was implemented by high-level research assistants with secondary school education. In routine care, similar interventions would likely be implemented by lower-level HCWs with multiple responsibilities, therefore, routine implementation may not see the same benefits. Second, there is always risk of social desirability bias in reporting acceptability of an intervention; however, qualitative researchers were not involved in implementation of HIVST, potentially minimizing any social desirability bias. Third, observational journals are based on events and conversations observed by study staff and cannot capture all events – mundane events may be particularly missed if staff deem them unworthy of documentation. Additional limitations of observational journals are discussed elsewhere [22]. Finally, the sample size of HIVST non-users is small, making it difficult to draw conclusions for this specific population. Additional research should be conducted with those in need of HIV testing but refuse HIVST.

## Conclusion

Facility HIVST in OPD waiting spaces was acceptable and feasible among adult outpatients in Malawi. Using waiting spaces for HIVST distribution and use, alongside private spaces for kit interpretation, facilitated HIV testing for most respondents. Strategies to promote privacy throughout the HIVST process, to improve disclosure of test results to HCWs, and to strengthen post-test counseling are important areas for future research.

## Data Availability

The datasets used and/or analyzed during the current study are available from the corresponding author on reasonable request.
